# Precise pancreatic cancer therapy through targeted degradation of mutant p53 protein by cerium oxide nanoparticles

**DOI:** 10.1186/s12951-023-01867-6

**Published:** 2023-04-01

**Authors:** Hao Zhang, Wang Zhang, Bochuan Hu, Xiaohua Qin, Tianxiang Yi, Yayi Ye, Xiaowan Huang, Yang Song, Zhenyu Yang, Jieying Qian, Yunjiao Zhang

**Affiliations:** 1 School of Medicine, South China University of Technology, Guangzhou, 510006 P. R. China; 2 School of Biomedical Sciences and Engineering, South China University of Technology, Guangzhou International Campus, Guangzhou, 511442 P. R. China; 3 National Engineering Research Center for Tissue Restoration and Reconstruction and Key Laboratory of Biomedical Engineering of Guangdong Province, South China University of Technology, Guangzhou, 510006 P. R. China

**Keywords:** mutp53 degradation, CeO_2_ nanoparticles (CeO_2_ NPs), Ubiquitination proteasome, Cancer therapy

## Abstract

**Background:**

In a significant proportion of cancers, point mutations of TP53 gene occur within the DNA-binding domain, resulting in an abundance of mutant p53 proteins (mutp53) within cells, which possess tumor-promoting properties. A potential and straightforward strategy for addressing p53-mutated cancer involves the induction of autophagy or proteasomal degradation. Based on the previously reported findings, elevating oxidative state in the mutp53 cells represented a feasible approach for targeting mutp53. However, the nanoparticles previous reported lacked sufficient specificity of regulating ROS in tumor cells, consequently resulted in unfavorable toxicity in healthy cells.

**Results:**

We here in showed that cerium oxide CeO_2_ nanoparticles (CeO_2_ NPs) exhibited an remarkable elevated level of ROS production in tumor cells, as compared to healthy cells, demonstrating that the unique property of CeO_2_ NPs in cancer cells provided a feasible solution to mutp53 degradation. CeO_2_ NPs elicited K48 ubiquitination-dependent degradation of wide-spectrum mutp53 proteins in a manner that was dependent on both the dissociation of mutp53 from the heat shock proteins Hsp90/70 and the increasing production of ROS. As expected, degradation of mutp53 by CeO_2_ NPs abrogated mutp53-manifested gain-of-function (GOF), leading to a reduction in cell proliferation and migration, and dramatically improved the therapeutic efficacy in a BxPC-3 mutp53 tumor model.

**Conclusions:**

Overall, CeO_2_ NPs increasing ROS specifically in the mutp53 cancer cells displayed a specific therapeutic efficacy in mutp53 cancer and offered an effective solution to address the challenges posed by mutp53 degradation, as demonstrated in our present study.

**Supplementary Information:**

The online version contains supplementary material available at 10.1186/s12951-023-01867-6.

## Background

The p53 protein that encodes by TP53 gene, is one of the most important tumor suppressors which regulates the transcription of a large number of downstream target genes that serves to cell proliferation, apoptosis, and metabolism [1, 2]. However, at least half of human cancers have been commonly observed a genetic mutation in the TP53 gene and this mutation rate can reach as high as 96 % in certain cancer subtypes such as high-grade serous ovarian carcinoma [3, 4]. There are more than 75 % of these mutations in TP53 that are missense mutations, which occur primarily in the DNA-binding domain and involve one amino acid change, including Y220C, G245C, R282W, R273H, R175H, R249S, and R248W, and lead to the high expression of full-length mutant p53 proteins (mutp53) that stably accumulate in cancer cells [5, 6]. In contrast to the wild-type p53 protein, the mutp53, in most cases, loses the ability to interact with the specific DNA-binding sequence and consequently cannot activate the p53 tumor suppressive transcription response due to loss of wild-type p53 activity [7, 8]. In addition, unlike other tumor suppressors which are frequently acquire loss of-function mutations, the highly stabilized mutant p53 proteins may confer unique gain-of-function (GOF) capability to actively promote tumorigenic events [9, 10], including enhanced tumor growth, invasion, metastasis and resistance to therapeutic drugs.

Based on the continued presence of mutp53 in human cancer and its critical role in driving tumorigenesis, therapy targeting mutp53 naturally becomes an attractive therapeutic strategy for p53-mutated cancer. Among of the multifarious reported strategies of targeting mutp53, mutp53 degradation is the most direct way targeting to eliminate mutp53 protein for cancer therapy. Over the past two decades, small molecules have been developed to elicit mutp53 degradation through either the proteasomal or autophagic pathways, such as statins [11], NSC59984 [12], Zn(II)-curc [13] and MCB-613 [14], which exhibited effective cancer therapeutic effects, indicating that the degradation of mutp53 was a feasible strategy for p53-mutated cancer treatment. Furthermore, to be hyper stabilized, mutp53 proteins interact with the Hsp70 and Hsp90 chaperone complex [15–17]. This stable interaction, to a large extent, protects the mutp53 proteins from degradation through their E3 ubiquitin ligases MDM2 and CHIP [18–20]. Small molecule Hsp90 inhibitors ganetespib and 17-AAG have been successfully shown a mark reduction of overall mutp53 level [21, 22], indicating that dissociation of mutp53 from the heat shock proteins was a potential tactic to elicit mutant p53 degradation. In addition, the stability of mutp53 protein is closely related to the redox state of the cells, and elevating oxidative state in the mutant p53 cells is another alternative approach to targeting mutp53 [23]. Recently, we have developed various nanoparticles [24, 25], which exerted a remarkable increase of intracellular reactive oxygen species (ROS), to achieve cancer treatment effect by efficiently inducing mutp53 degradation, indicating that regulating ROS specifically in tumor cells was expected to solve the problem of mutp53 degradation. However, the nanoparticles previous reported as the mutp53 degraders identified so far oftentimes lacked sufficient specificity of elevating ROS in tumor cells, leading unfavorable toxicity in normal cells.

**Scheme 1:**
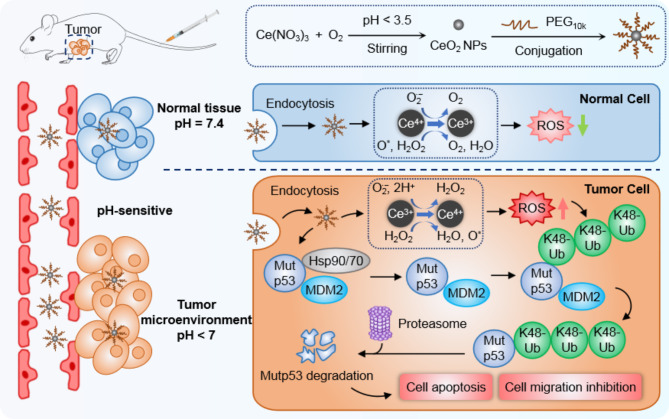
Schematic illustration for the synthesis of CeO_2_ NPs, nanoparticle internalization, ROS generation, dissociation of mutp53 from the heat shock proteins Hsp90/70 and UPS dependent mutp53 degradation in cancer

In recent years, by the excellent redox-modulatory and enzyme-like activities, cerium oxide (CeO_2_) nanoparticles are envisaged as promising candidates in nanomedicine when they come to a number of applications, including cancer treatments [26, 27]. An increasing studies have been reported that CeO_2_ NPs have pro-oxidant properties in the acidic pH environment of cancerous cells, exhibiting an significant-elevated level of ROS production, while they have antioxidant properties in the neutral pH environment of healthy cells [28]. Thus, CeO_2_ NPs possesses both cytotoxic and protective properties that make them be the powerful agents for making cancer treatments more effective. And this unique property of CeO_2_ NPs is exactly consistent with our strategy to degrade mutp53 by increasing intracellular ROS production, indicating that CeO_2_ NPs present a perfect choice to specifically targeting the mutp53-expressing cancer. Here in, our findings on CeO_2_ NPs firstly revealed that CeO_2_ NPs were significantly more cytotoxic to mutp53 cancer cells than to wild-type p53 cancer cells, which encouraged us to explore further intrinsic mechanism into the possible targeting ability of CeO_2_ NPs on mutp53. Our results demonstrated that CeO_2_ NPs could efficiently elicit mutp53 degradation but not wild type p53 protein. To gain more a proof, we investigated that CeO_2_ NPs was successfully able to induce K48 ubiquitination-dependent degradation for a panel of mutp53s in a manner that was dependent on both heat shock proteins Hsp90/70 dissociation from the mutp53 and increasing ROS formation. There was also compelling evidence that the degradation of mutp53 caused by CeO_2_ NPs resulted in abrogation of the multiple mutp53-based GOFs, and this in turn led to substantial therapeutic effectiveness in BxPC-3 mutp53 pancreatic tumor model. Our work therefore revealed the CeO_2_ NPs exerted an outstanding and specific therapeutic efficacy in mutp53 cancer and provided an alternative way to address the challenges posed by mutp53 degradation (Scheme 1).

## Results and discussion

### Synthesis, characterization of CeO_2_ NPs

**Fig. 1:**
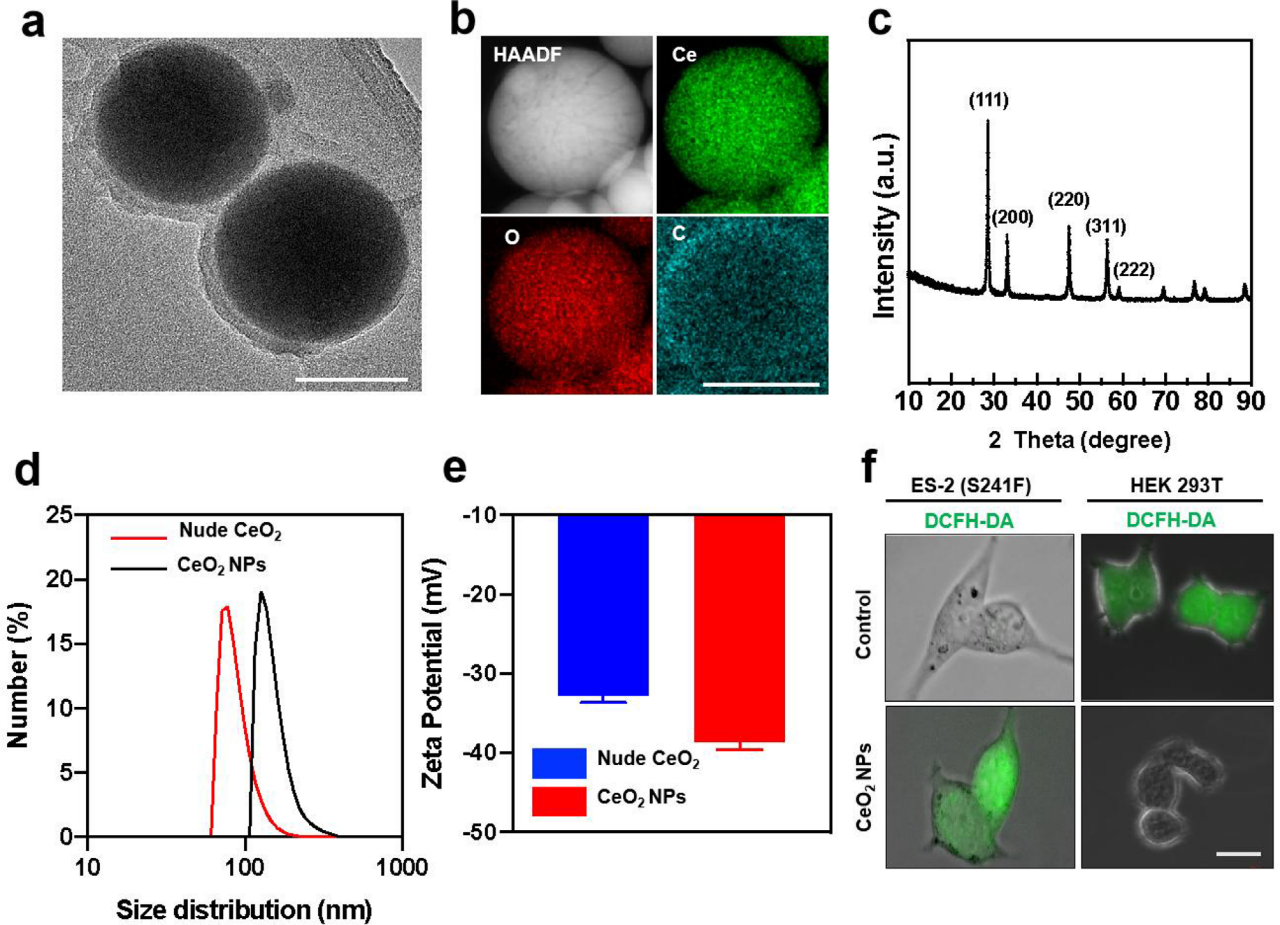
**Characterization of the structural properties of CeO**_**2**_**NPs.(a)** TEM image of CeO_2_ NPs. Scale bar, 100 nm. **(b)** HAADF-STEM image of CeO_2_ NPs, together with the corresponding element maps from EDX for Ce and O. Scale bar, 100 nm. **(c)** XRD pattern of CeO_2_ NPs. **(d-e)** Size distribution in water **(d)** and the Zeta potential **(e)** analysis of nude CeO_2_ NPs and CeO_2_ NPs. **(f)** The levels of intracellular ROS were detected with DCFH-DA and analyzed by fluorescence imaging after treatment with PBS (Control) or CeO_2_ NPs (6 µg mL^− 1^) for 4 h. Scale bar, 20 μm

A simple wet chemistry method was used to synthesize the CeO_2_ NPs following the previous publications with slight modification. Briefly, a stoichiometric amount of cerium nitrate hexahydrate was stirring in the solution for 1 h with pH below 3.5, which allowed the cerium (III) ions in the solution to be oxidized to cerium (IV) oxide. The oxidation of CeO_2_ NPs resulted in the formation of crystalline nanoparticles. Finaly, CeO_2_ NPs conjugated with PEG10 K in order to enhance their biocompatibility and water solubility. The transmission electron microscopy (TEM) images showed that the CeO_2_ NPs were spherical with an average size of approximately 100 nm (Fig. 1a). Moreover, high-resolution TEM (HRTEM) of CeO_2_ NPs was shown in **Figure **S1a. It was also consistent with the SAED pattern (**Figure **S1b) to measure the lattice fringe spacing of the samples at 0.26 nm. Furthermore, a high-angle annular dark-field scanning TEM (HAADF-STEM) together with energy-dispersive X-ray spectroscopy (EDX) was performed to investigate the structures of CeO_2_ NPs and the results demonstrated that Ce (green) and O (red) are distributed evenly in the CeO_2_ NPs, which further confirmed that they had been synthesized successfully (Fig. 1b). X-ray diffraction (XRD) and X-ray photoelectron spectrometry (XPS) were carried out to reveal the crystalline phases of the CeO_2_ NPs. As can be seen from Fig. 1c, the XRD pattern showed the diffraction peak of the prepared nanomaterials, which was in agreement with the JCPDS file for CeO_2_ NPs (JCPDS 34–394). And the XPS measurements were employed as a method of further confirmation. In order to calibrate these binding energies, the C (1s) peak at 275.6 eV was used as a reference. Noticeably, the peaks at 918.6 and 884.5 eV have been assigned to Ce 3d 3/2 and Ce 3d 5/2, respectively (**Figure **S2a). And satellite peaks or vibration peaks were not to be observed, which was in accordance with + 4 valent Ce. The peaks at 529.4 eV, on the other hand, were indicative of the binding energy of O1S (**Figure **S2b), which suggested the presence of a -2 valent O. And the figure showed a typical survey spectrum of CeO_2_ NPs as illustrated in **Figure **S2c. These results proved the highly crystalline of the CeO_2_ NPs we synthesized. Besides, we have carried out dynamic light scattering (DLS) and zeta potential experiments to detect the size and charge of nude CeO_2_ NPs and CeO_2_ NPs (PEG-modification) in aqueous solution. The results showed an average hydrated size of 88 nm of nude CeO_2_ NPs in water, and the zeta potential of nude CeO_2_ NPs was around − 33 mV, while the size and zeta potential of PEGylated CeO_2_ NPs have exhibited 150 nm and − 38mV respectively (Fig. 1d and e), consistent with published reports [29, 30]. In addition to test the dispersion and stability of the CeO_2_ NPs in aqueous solution, DLS was also conducted over a sustained period of time and the result showed an average hydrated size of 150 nm of CeO_2_ NPs (**Figure **S3), indicating a long-term storage of CeO_2_ NPs and thus meet the demands for the application in biomedicine. The above characterization results of CeO_2_ NPs indicate that we have successfully synthesized CeO_2_ NPs. To confirm the pro-oxidant properties and antioxidant properties in cancer cells and normal cells respectively, ES-2 ovarian cancer cell line (mutp53 cancer cell) and HEK 293T (normal cell) were treated with CeO_2_ NPs at the equal mass concentration of 6 µg mL^− 1^ for 4 h. As expected, CeO_2_ NPs significantly increased the intracellular reactive oxygen species (ROS) in ES-2 cells, as revealed by fluorescent imaging, following by staining with DCFH-DA, while CeO_2_ NPs had a opposite effect on the intracellular ROS in HEK 293T cells (Fig. 1f), consistent with the reported study that CeO_2_ NPs increased the production of ROS specifically in cancer cells.

## CeO_2_ NPs triggered remarkable degradation of wide spectrum mutp53

**Fig. 2:**
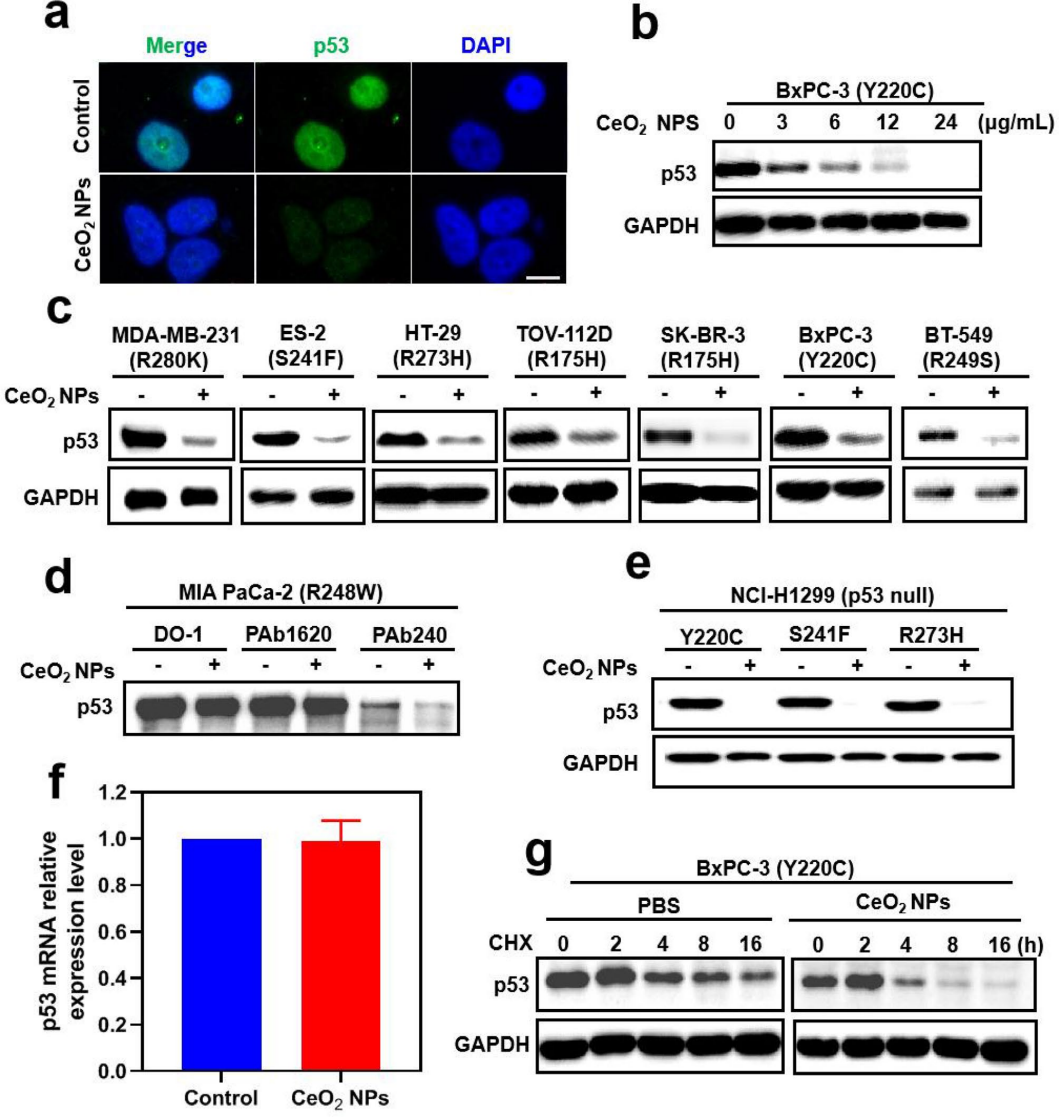
**CeO**_**2**_**NPs induced the degradation of wide spectrum mutp53. (a)** Confocal microscopic images of BxPC-3 cells treated with PBS (Control) or CeO_2_ NPs (6 µg mL^− 1^) for 12 h, followed by immunostaining with p53 antibody and nucleus staining with DAPI. Scale bar, 10 μm. **(b)** Western blotting of p53 in BxPC-3 cells treated with CeO_2_ NPs for the indicated doses for 12 h. **(c)** Western blotting analysis of p53 was performed on p53 mutants (R280K, S241F, R273H, R175H, Y220C and R249S) endogenously expressed in MDA-MB-231, ES-2, HT-29, TOV-112D, SK-BR-3, BxPC-3 and BT-549 cell lines, respectively, following treatment with CeO_2_ NPs (6 µg mL^− 1^) for 12 h. **(d)** Immunoprecipitation (IP) analysis was conducted for total p53 (DO-1), the folded/native form of p53 (PAb1620), and the misfolded/denatured form of p53 (PAb240), using MIA PaCa-2 cells that were treated with PBS or CeO_2_ NPs (6 µg mL^− 1^) for 12 h. **(e)** Western blotting analysis of p53 in NCI-H1299 cells transfected with Y220C, S241F and R273H mutp53-expressing plasmid and then treated with CeO_2_ NPs (6 µg mL^− 1^) for 18 h. **(f)** Quantitative RT-PCR analysis of the relative level of p53 mRNA in BxPC-3 cells treated with PBS (Control) or CeO_2_ NPs (6 µg mL^− 1^) for 12 h. Mean ± s.e.m. *n* = 3. **(g)** Western blotting analysis was performed on BxPC-3 cells treated with cycloheximide (CHX, 50 µM) either in the absence or presence of CeO_2_ NPs (6 µg mL^− 1^) for the indicated hours

As described above, we want to evaluate the potential mutp53-degrading effect of CeO_2_ NPs. We firstly carried out immunofluorescence analysis in BxPC-3 pancreatic carcinoma harboring Y220C mutation and the results showed that CeO_2_ NPs significantly reduced mutp53 levels compared to those in the control groups (Fig. 2a). The same phenomenon was observed in ES-2 cells (**Figure **S4). The reduced mutp53 protein level was further confirmed by western blotting and revealed that CeO_2_ NPs markedly decreased the level of mutp53 proteins in both dose- and time-dependent fashion (Fig. 2b, S5), with significant reduction of mutp53 in BxPC-3 cells beginning to be observed at 12 h with the concentration of 6 µg mL^− 1^, indicating the remarkable degradation of mutp53 by CeO_2_ NPs. In addition to Y220C mutp53, CeO_2_ NPs also notably decreased the level of several other endogenous mutp53 in multiple tumor cell lines, including MDB-MA-231 (R280K), ES-2 (S241F), HT-29 (R273H), TOV-112D (R175H), SK-BR-3 (R175H) and BT-549 (R249S) (Fig. 2c). A noteworthy finding was that of six mutations that were efficiently depleted by CeO_2_ NPs, and three mutants (R280K, R273H and S241F) were corresponded to DNA contact mutants, while the remaining three (Y220C, R249S and R175H) were conformational mutants. These results showed that CeO_2_ NPs had no preference for a particular class of mutations. Due to this characteristic, CeO_2_ NPs stand out from other known mutp53 eliminators that are known for preferring mutp53s with conformational defects. In contrast, CeO_2_ NPs had minimal effect on the level of wild-type p53 protein in the A549 cell line (**Figure **S6a, S6b), suggesting that mutp53 was selectively degraded by CeO_2_ NPs. To lend further proof that the CeO_2_ NPs could specifically induced degradation of mutp53, MIA PaCa-2 cells (R248W) was used as a system for immunoprecipitation experiment. According to the publication that there was a folded/native conformation of p53 in the R248W mutant [11], as well as a misfolded/denatured conformation, which could be detected with conformation-specific antibodies PAb1620 and PAb240, respectively. Based on the results presented in Fig. 2d, it was demonstrated that CeO_2_ NPs was primarily depleting p53 (R248W) in its misfolded form. Moreover, CeO_2_ NPs was also able to significantly deplete mutp53 exogenously expressed in the p53-null NCI-H1299 cells by depleting the mutp53 Y220C, mutp53 R273H as well as the mutp53 S241F, notwithstanding after a longer period of treatment by CeO_2_ NPs (18 h) (Fig. 2e).

To explore the specific mechanism by which CeO_2_ NPs depleted mutp53, we tested the BxPC-3 cells using RT-PCR in order to determine whether the reduced level of mutp53 was the result of decreased transcription. TP53 mRNA levels were not affected after CeO_2_ NPs treatment, indicating that no alterations in TP53 transcript levels were observed after CeO_2_ NPs treatment (Fig. 2f). However, we observed a significant decrease in the level of mutp53 when cycloheximide was combined with CeO_2_ NPs compared to cycloheximide treatment alone (Fig. 2g). According to the above experimental results, CeO_2_ NPs induced degradation of mutp53 at the post-translational level.

## CeO_2_ NPs degraded mutp53 in a proteasome-dependent manner

**Fig. 3:**
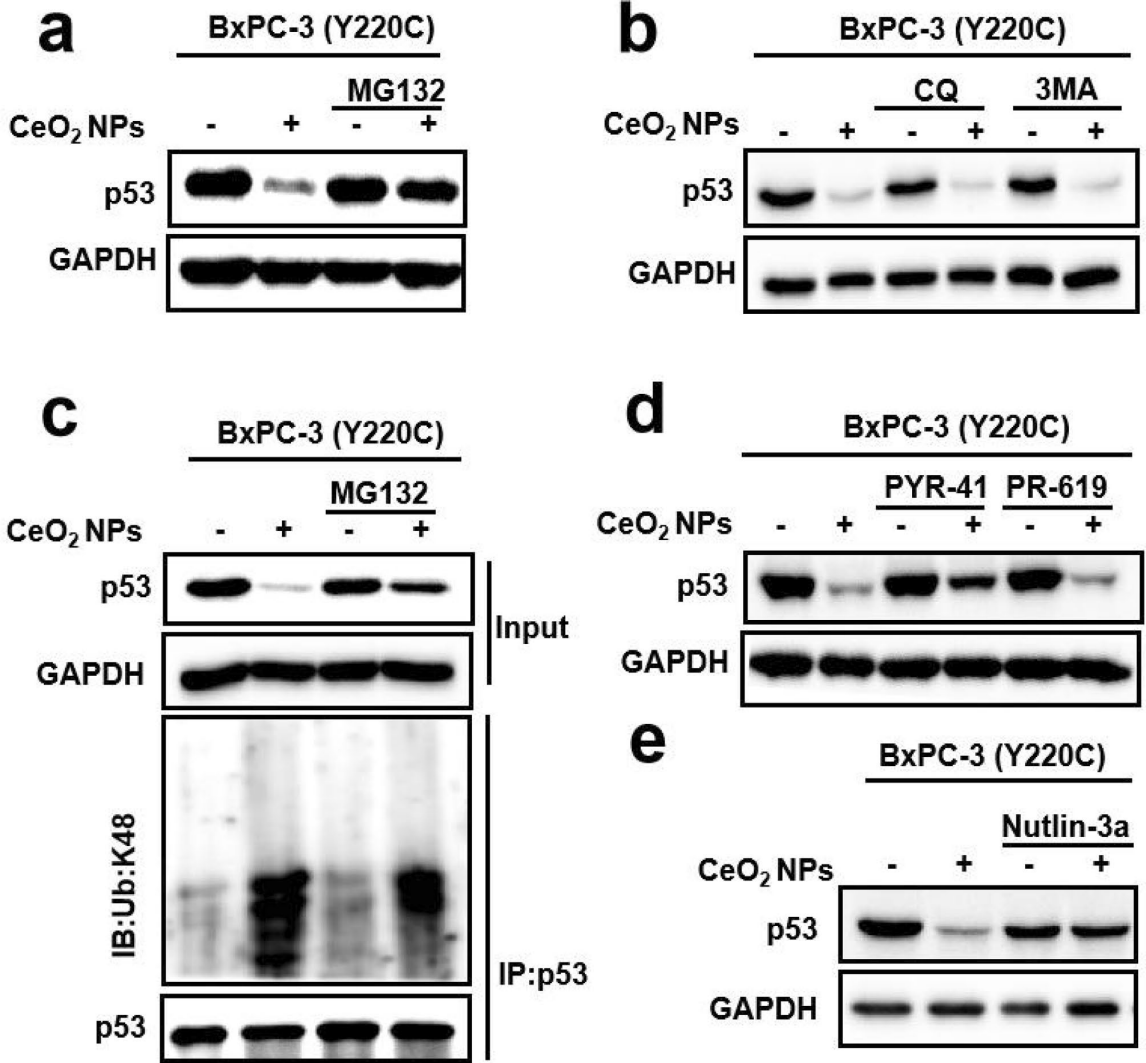
**CeO**_**2**_**NPs degraded mutp53 through ubiquitin proteasome pathway. (a,b)** Western blotting analysis of p53 in BxPC-3 cells after treatment with the indicated substances for 12 h. Dosing: CeO_2_ NPs (6 µg mL^− 1^); CQ, 50 µM; 3-MA, 5 mM; MG132, 10 µM. **(c)** Immunoprecipitated p53 was analysed using K48-ub in BxPC-3 cells that had been treated with CeO_2_ NPs (6 µg mL^− 1^), MG132 (10 mm) or CeO_2_ NPs + MG132 for 12 h. **(d)** Western blotting analysis of p53 levels in BxPC-3 cells after the indicated treatment for 12 h. Dosing: PYR-41, 50 µM; CeO_2_ NPs, 6 µg mL^− 1^; PR-619, 5 µM. **(e)** Western blotting analysis of p53 in BxPC-3 cells after treatment with the indicated substances for 12 h. Dosing: CeO_2_ NPs, 6 µg mL^− 1^; Nutlin-3a, 10 µM

In order to investigate the mechanism for the degradation of mutp53 by CeO_2_ NPs, we examined both proteasome and autophagy, which are the two major cellular pathways for protein degradation. MG132, an inhibitor of proteasomal degradation, completely abolished the mutp53 degradation in BxPC-3 cells caused by CeO_2_ NPs (Fig. 3a), similar phenomenon also being observed in ES-2, MIA PaCa-2, and HT-29 cells (**Figure **S7), but the autophagy inhibitors 3-MA and chloroquine (CQ) had a minimal effect on the reduction of mutp53 induced by CeO_2_ NPs (Fig. 3b). This result indicated that the degradation of mutp53 induced by CeO_2_ NPs was dependent on the proteasome pathway rather than the autophagy pathway. Moreover, the ubiquitin system exerted an essential role in specific recognition and degradation of proteasome substrate proteins, and the polyubiquitination modification at K48 was usually used as the degradation signal of proteasome. Therefore, we wanted to investigate whether the ubiquitination level of mutp53 was increased during CeO_2_ NPs inducing degradation of mutp53. A result in BxPC-3 cells demonstrated that the presence of CeO_2_ NPs enhanced K48 polyubiquitination in immunoprecipitated p53 (Fig. 3c). Besides, the protein ubiquitin activating enzyme E1 (UBA1) inhibitor PYR-41 was also shown to be able to effectively inhibited CeO_2_ NPs-induced degradation of mutp53 in BxPC-3 cells, but the deubiquitinylating enzyme inhibitor PR-619 had a reverse effect, promoting the degradation of mutant p53 induced by CeO_2_ NPs (Fig. 3d). Based on these results, it could be concluded that CeO_2_ NPs degraded mutp53 protein through a ubiquitination-proteasomal pathway. As described above, in ubiquitination proteasomal system, highly conserved E3 ligases specifically recognize targeting substrates proteins, and we have tested MDM2, which is a major E3 ligase candidate of p53 protein. The result showed that the MDM2 inhibitor nutlin-3a was able to effectively inhibit the CeO_2_ NPs-induced degradation of mutp53 in BxPC-3 cells (Fig. 3e), exhibiting a similar phenomenon in MIA PaCa-2 cells (**Figure **S8), suggesting that MDM2 ubiquitin E3 ligase was responsible for mutp53 degradation caused by CeO_2_ NPs. These results suggested that the degradation of mutp53 induced by CeO_2_ NPs was dependent on the ubiquitination-proteasome pathway mediated by the ubiquitin E3 ligase MDM2.

## A combination of nanoparticle internalization, heat shock protein Hsp90/70 dissociation, and increasing intracellular ROS was essential for the mutp53 degradation by CeO_2_NPs

**Fig. 4:**
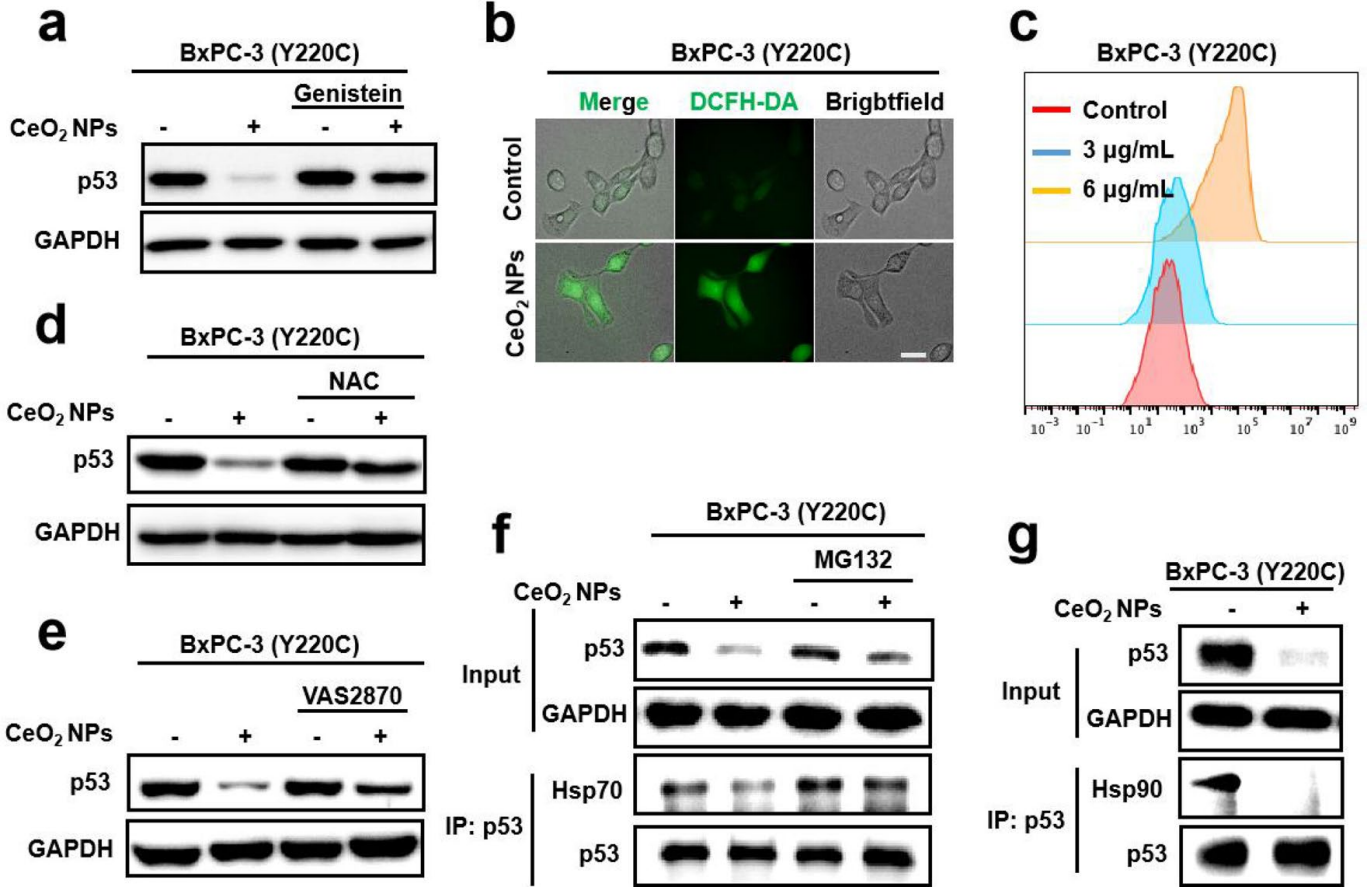
**Cellular internalization, heat shock protein Hsp90/70 dissociation and ROS were all required for CeO**_**2**_**NPs-elicited mutp53 degradation**. **(a)** Western blotting analysis of p53 levels in BxPC-3 cells after indicated treatments for 12 h. Dosing: Genistein, 50 µM; CeO_2_ NPs, 6 µg mL^− 1^. **(b-c)** The levels of intracellular ROS were detected with DCFH-DA and analyzed by immunofluorescence (b) or flow cytometer analysis (c) after treatment with PBS (Control) or CeO_2_ NPs (3 or 6 µg mL^− 1^) for 4 h. Scale bar, 10 μm. **(d)** Western blotting analysis of p53 levels in BxPC-3 cells after treatment with the indicated treatment for 12 h. Dosing: NAC, 5 mM; CeO_2_ NPs,6 µg mL^− 1^). **(e)** Western blotting analysis of p53 levels in BxPC-3 cells after indicated treatments for 12 h. Dosing: CeO_2_ NPs, 6 µg mL^− 1^; VAS 2870, 15 µM. **(f)** Western blotting of Hsp70 and p53 for the immunoprecipitated p53 in BxPC-3 cells treated with CeO_2_ NPs (6 µg mL^− 1^), MG132 (10 µM) or CeO_2_ NPs + MG132 for 12 h. **(g)** Western blotting analysis of Hsp90 and p53 for the immunoprecipitated p53 in BxPC-3 cells after CeO_2_ NPs treatment (6 µg mL^− 1^) for 12 h

To further explore the specific molecular mechanism of CeO_2_ NPs-induced degradation of mutp53, we first conducted an experiment in which we co-treated CeO_2_ NPs with genistein, a molecule that inhibited caveolae-mediated endocytosis, to determine whether endocytosis was necessary for mutp53 degradation induced by CeO_2_ NPs. There was a significant suppression of mutp53 degradation by genistein when CeO_2_ NPs was applied (Fig. 4a), and the quantization result was displayed in the Figure S9, suggesting that CeO_2_ NPs must be internalized to the cells before mutp53 degradation. As mentioned above, CeO_2_ NPs could trigger a significant increasing intracellular ROS level in ES-2 cells (Fig. 1f), and similar results were obtained in BxPC-3 and MIA PaCa-2 cells, as revealed by both fluorescent imaging (Fig. 4b) and flow cytometer analysis (Fig. 4c, **Figure **S10), following the addition of DCFH-DA treatment, which detected the total level of intracellular ROS in the cells. These experiments demonstrated that CeO_2_ NPs could induce ROS generation in mutp53 tumor cells. To explore the relationship between CeO_2_ NPs-induced degradation of mutp53 and the elevation of ROS levels, we used N-acetyl cysteine (NAC), an effective free radical scavenger, co-treated with CeO_2_ NPs and the results showed that NAC could effectively abolished the ability of CeO_2_ NPs to degrade mutp53 in BxPC-3 cells, consistent with the previous publications (Fig. 4d). A similar phenomenon was observed in MIA PaCa-2 and TOV-112D cells as well (**Figure **S11). Moreover, VAS 2870, an inhibitor of NADPH oxidase, significantly inhibited CeO_2_ NPs-induced degrading of mutp53 (Fig. 4e). These results demonstrated that intracellular elevation ROS exerted an important role in degradation of mutp53 in tumor cells induced by CeO_2_ NPs. Additionally, many publications revealed that mutp53 proteins in cancer cells formed stable complexes with the heat shock protein 90 and heat shock protein 70, which promoted mutp53 largely and stably accumulated in tumor cells and failed to degrade in ubiquitination proteasomal system. Immunoprecipitation assay was used to investigate whether CeO_2_ NPs treatment affected the interaction between mutp53 and Hsp90 or Hsp70, resulting in reduced stability and degradation of mutp53 protein. It was observed that CeO_2_ NPs treatment led a reduction in the level of interaction between mutp53 and Hsp90 or Hsp70 (Fig. 4f g), suggesting that CeO_2_ NPs induced the dissociation of mutp53 from Hsp90 or Hsp70, thereby affecting the stability of mutp53 protein. To lend further proof, PLA assay was also carried out to investigate the effect of CeO_2_ NPs treatment on the interaction between mutp53 and Hsp90. A significant reduction in the Hsp90-mutp53 PLA signal was observed in the BxPC-3 cell after CeO_2_ NPs treatment (**Figure **S12), which further demonstrated that the interaction between mutp53 and Hsp90 was reduced by CeO_2_ NPs treatment. These data suggested that the separation of Hsp90/Hsp70 from mutp53 was essential for CeO_2_ NPs-induced degradation of mutp53.

Based on the results outlined above, it can be concluded that cellular internalization, dissociation of the Hsp 90/70 from mutp53 and the production of reactive oxygen species are all required for the degradation of the mutp53 protein induced by CeO_2_ NPs.

## The degradation of mutp53 by CeO_2_ NPs impaired GOF phenotypes derived from mutp53

**Fig. 5:**
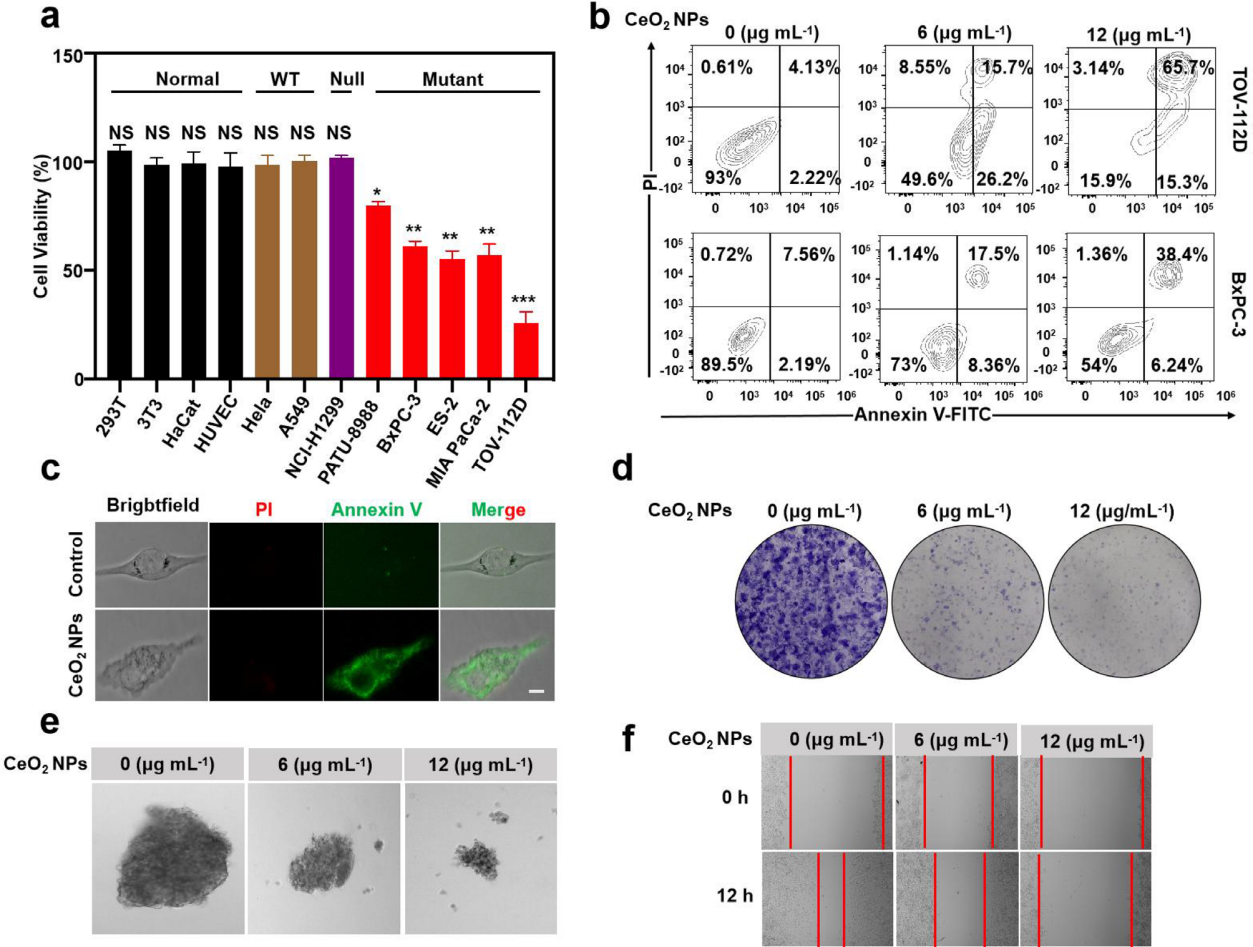
**Degradation of mutp53 by CeO**_**2**_**NPs impaired mutp53-conferred GOF phenotypes(a)** MTT assay of normal cells (HEK 293T, 3T3, HaCat and HUVEC), wild type p53 cells (A549, Hela), p53 null cells (NCI-H1299) and mutp53 (PATU-8988, BxPC-3, ES-2, MIA PaCa-2 and TOV-112D) cells treated with PBS or CeO_2_ NPs (6 µg mL^− 1^) for 12 h. Mean ± s.e.m. *n* = 3, ***p < 0.001. **p < 0.01. *Student’s t-test.***(b)** Assays of annexin-V/PI on mutp53 (TOV-112D and BxPC-3) cells treated with PBS or CeO_2_ NPs (6 or 12 µg mL^− 1^) for 12 h. **(c)** Fluorescence microscopy images of ES-2 cells stained with Annexin-V/PI after PBS (Control) or CeO_2_ NPs (6 µg mL^− 1^) for 12 h. **(d)** Clonal formation assay of BxPC-3 cells after treatment with PBS or CeO_2_ NPs (6 or 12 µg mL^− 1^) for 12 h. **(e)** Sphere-forming assay of BxPC-3 cells treated with PBS or CeO_2_ NPs (6 µg mL^− 1^ or 12 µg mL^− 1^) for 12 h. **(f)** Cell migration assay of BxPC-3 cells treated with PBS (0 µg mL^− 1^) or CeO_2_ NPs (6 or 12 µg mL^− 1^) for 12 h

The highly stable expression and accumulation of mutp53 protein played a crucial role in promoting the growth, survival and metastasis of cancer cells. Therefore, degradation of mutp53 by CeO_2_ NPs would be expected to exhibit higher toxicity in mutp53 cells than in p53 wild-type or normal cells. As expected, CeO_2_ NPs treatment resulted in a significant decrease in cell viability in five mutp53 cell lines, including PATU-8988, BxPC-3, ES-2, MIA PaCa-2 and TOV-112D, and even the viability-reduction reached 75% in TOV-112D cells, whereas it had little effect on the cell viability of the two cell lines expressed wild type p53 proteins (Hela and A549) and the p53 null cell line (NCI-H1299), as well as four normal cell lines (HEK 293T, 3T3, HaCat, and HUVEC) we tested (Fig. 5a). These results indicated that CeO_2_ NPs specifically killed the tumor cells expressing mutp53 protein, but not wild-type p53 and normal cells. At the same time, in order to explore the effect of CeO_2_ NPs induced degradation of mutp53 on apoptosis in mutp53 cell lines, Annexin-V/PI staining assay was performed. It was found that mutp53 tumor cells showed an increased level of apoptotic cells revealed by flow cytometer analysis following Annexin-V/PI staining (44% and 81% for BxPC-3 and TOV-112D cells respectively with the concentration of 12 µg mL^− 1^ of CeO_2_ NPs) (Fig. 5b). A similar Annexin-V/PI fluorescence staining result was observed in the ES-2 cell line as well (Fig. 5c). The above experimental results indicated that CeO_2_ NPs elicited significant apoptosis in mutp53 cells through the degradation of mutp53 induced by CeO_2_ NPs. In order to further explore the effect of CeO_2_ NPs induced degradation of mutp53 on GOF of tumor cells, we carried out clonal formation assay, cell migration assay and sphere-formation assay. As expected, degradation of mutp53 in BxPC-3 cells by CeO_2_ NPs gave rise to abrogation of GOF phenotypes, characterized by decreased colony formation (Fig. 5d) and sphere-formation  (Fig. 5e). What’s more, it was also found that the cell-migration efficiency was dropped after CeO_2_ NPs treatment, when compared to the control (Fig. 5f). Based on the above results, we concluded that CeO_2_ NPs selectively destroyed mutp53 cells by abolishing the GOF of mutp53.

## CeO_2_ NPs exerted an effective cancer therapeutic efficacy in BxPC-3 pancreatic cancer model

**Fig. 6:**
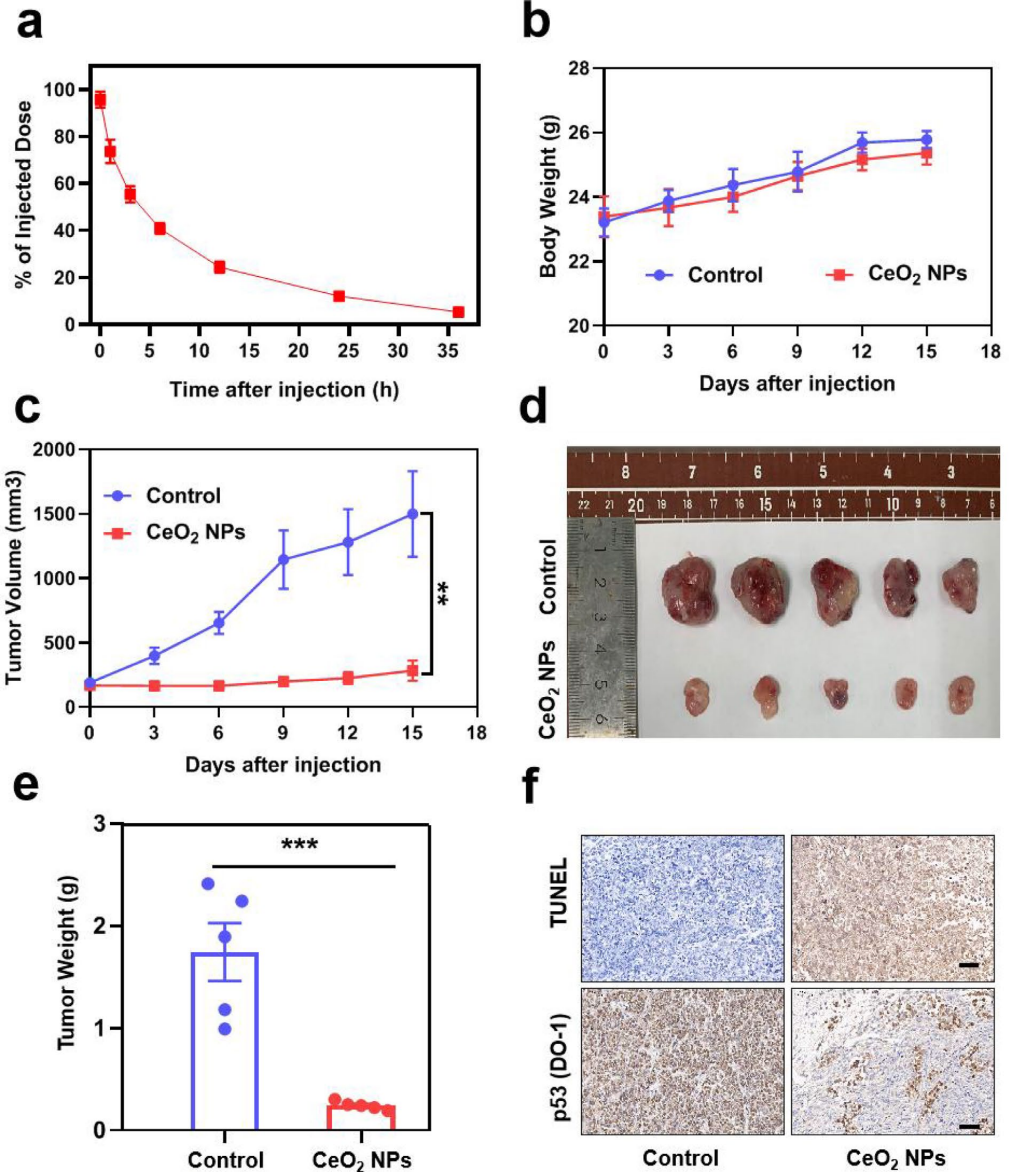
**CeO**_**2**_**NPs elicited excellent therapeutic efficacy in the BxPC-3 pancreatic cancer model**. **(a)** Pharmacokinetic profile of Ce after intravenous injection at a dose of 30 mg kg^− 1^ of CeO_2_ NPs. **(b,c)** Change in the body weight of mice (b) and the tumor volume (c) during the 15-day therapeutic period in the BxPC-3 model in nude mice. Dosing: 30 mg kg^-1^ CeO_2_ NPs, IV injection twice a week. **(d)** Images of the tumors excised from the mice on day 15 in the various treatment groups. **(e)** Tumor weight of mice on day 15 in the various treatment groups. Mean ± s.e.m. n = 5. ***p < 0.001. *Student’s t-test.***(f)** Sections of the tumor were stained with TUNEL staining and immunohistochemical staining of p53 on the excised tumor tissues from the different treatment groups in the BxPC-3 model. Scale bar, 50 μm

The previous experiments have explored and verified the specific molecular mechanism of CeO_2_ NPs-induced degradation of mutp53 and demonstrated CeO_2_ NPs selectively destroyed p53-mutated tumor cells by eliminating the GOF of mutp53. And then we would like to investigate the cancer therapeutic effect of CeO_2_ NPs in animal model. As a prelude to cancer therapeutic study, we carried out preliminary evaluation on the pharmacokinetics and bio-safety of the CeO_2_ NPs. As shown in Fig. 6a, it revealed the metabolism of CeO_2_ NPs in the bloodstream following intravenous administration. Furthermore, we have also conducted HE staining on major organs (heart, liver, spleen, lung and kidney) for histological analysis and found no histological differences in these tissues between control (PBS) and CeO_2_ NPs treatment groups, indicating no notable toxicity (**Figure **S13). At the same time, we checked aspartate aminotransferase (AST) and alanine aminotransferase (ALT) to assess liver function, creatinine (SCR) and blood urea nitrogen (BUN) to evaluate kidney activity by biochemical analysis. We found no obvious changes in these key biochemical markers in serum from mice after CeO_2_ NPs treatment (**Figure **S14), further confirming the bio-safety of CeO_2_ NPs for application *in vivo*.

To assess the anti-tumor efficacy of CeO_2_ NPs mediated degradation of mutp53 *in vivo*, we established a BxPC-3 pancreatic tumor model in nude mice. We carried out a 15-day twice-a-week treatment regimen under the guidance of an intravenous injection of 30 mg kg^− 1^ CeO_2_ NPs, starting at a point which the tumor volume reached an approximate volume of 100 ~ 150 mm^3^. During the course of the therapeutic period, no significant differences in body weight have been observed across the treatment groups, which was in accordance with the results of the toxicity evaluation (Fig. 6b). Compared to the PBS group, CeO_2_ NPs significantly inhibited tumor growth (Fig. 6c). In agreement, the tumor weights were well consistent with the tumor volume data, with CeO_2_ NPs treatment eliciting a dramatic reduction in tumor weight. Importantly, we observed a dramatic reduction of the tumor size up to 86 % for CeO_2_ NPs treatment, indicating the excellent anti-tumor efficacy of CeO_2_ NPs (Fig. 6d and e). TUNEL assay of tumor sections also further confirmed the anti-tumor efficacy of CeO_2_ NPs, with increasing cell apoptosis upon treatment with CeO_2_ NPs (Fig. 6f). To verify CeO_2_ NPs exerted mutp53 degradation i*n vivo*, we also assessed p53 expression level in tumor sections obtained at the end of the treatment period evaluated by immunohistochemistry analysis and western blotting. In agreement with the results observed *in vitro*, the experimental groups treated with CeO_2_ NPs effectively decreased mutp53 expression level in tumor sections, suggesting that CeO_2_ NPs also elicited excellent mutp53 degradation *in vivo* (Fig. 6f,S15). Accordingly, the above results indicated that CeO_2_ NPs had the superior ability to produce anti-cancer effects when applied to the BxPC-3 pancreatic cancer model as a whole.

## Conclusion

In summary, we have demonstrated that CeO_2_ NPs exhibited remarkably activity toward degradation of a panel of mutp53 proteins, but not the wild-type p53. Cellular internalization of the CeO_2_ NPs, enhancement of ROS generation and dissociation of the heat shock proteins Hsp90/70 were all required for CeO_2_ NPs-induced mutp53 degradation through ubiquitination-mediated proteasome pathway. Moreover, mutp53-mediated GOF was abrogated by CeO_2_ NPs treatment, which resulted in diminished cell proliferation and cell migration of cancer cells with mutp53. Additionally, a subcutaneous BxPC-3 tumor model with mutp53 depleted by CeO_2_ NPs also showed dramatic improvements in therapeutic efficacy when exposed to CeO_2_ NPs. More importantly, CeO_2_ NPs promoting the production of ROS selectively in the mutp53 cancer cells revealed in our study provided a perfect choice to mutp53 degradation.

## Materials and methods

### Conjugation method of PEGylated CeO_2_

To synthesize PEGylated CeO_2_ NPs, CeO_2_ nanoparticles (15 mg) were mixed with Milli-Q water (6 mL), and then mPEG-NH_2_ (80 mg) was added into the solution followed by sonicating for 20 min and stirring overnight. Subsequently, the solution was centrifugalized with 12000 rpm for 25 min, and the free mPEG-NH_2_ was removed by washing with Milli-Q water for three times. The final products were obtained by lyophilization and finally resuspended with Milli-Q water for further experiments.

### Cell lines

DMEM or RPMI 1640 medium was used to maintain various human cell lines (which differed in their p53 status): BxPC-3、ES-2、NCI-H1299、MIA PaCa-2、TOV-112D、MDA-MB-231, HT-29, BT-549, SK-BR-3, A549, Hela, HEK 293T, 3T3, HaCat, HUVEC and PATU-8988. All cell lines we used in this study were from ATCC, and all of the cells were grown at 37 °C under 5 % CO_2_.

### Chemicals and compounds

The following chemicals were obtained from Selleck Chemicals: 3-methyladenine, PR-619, Cycloheximide, VAS 2870, MG132, PYR-41, and CQ. The NAC and Genistein were both purchased from Sigma-Aldrich.

### Western blotting (WB)

The cells were cultured in 24-well plate, followed by treatment with CeO_2_ NPs for 12 h. After treatments, the cells were lysed with phosphatase and protease-containing radioimmunoprecipitation assay (RIPA) buffer to prepare the samples for WB. The lysates of 100 mg of protein were concentrated on 13.5 % tris-glycine gels, separated by electrophoresis and applied to nitrocellulose membranes (NC), followed by incubation with primary antibodies specific to the protein of interest and secondary antibodies conjugated with HRP. The GE Amersham Imager imaging system was used as a method to analyze all blots. These experiments were conducted using the following antibodies: anti-p53 (sc-126, DO-1, 1:1000 dilution, Santa Cruz Biotechnology), anti-K48-Ub (12805s, 1:2000 dilution, Cell Signaling Technology) and anti-GADPH (60004-1-Ig, 1:2000 dilution, Proteintech).

### Co-immunoprecipitation

The cells were lysed in IP lysis buffer with a protease inhibitor cocktail. Approximately 250 µg of whole-cell lysates were incubated with 1 µg antibodies (PAb1620,OP33, Millipore; PAb240, sc-99, Santa Cruz Biotechnology; sc-126, DO-1, Santa Cruz Biotechnology) overnight at 4 °C and then precipitated with protein A/G-agarose beads (sc-2003, Santa Cruz Biotechnology). Using antibodies against p53 (ab17990, Abcam), Hsp90 (60318-1-Ig, Proteintech), and Hsp70 (66183-1-Ig, Proteintech), we resolved the precipitates on SDS-PAGE followed by western blotting (WB).

### Ubiquitination analysis of mutp53

The cells were cultured in 6-well plate, followed by treatment with CeO_2_ NPs for 12 h. After treatments, an NP40 lysis buffer solution with the protease inhibitor cocktail (C600387, Sangon) was used to lyse cells. Cell extracts were incubated with the protein A/G-Agarose and the p53 antibody (sc-126, Santa Cruz Biotechnology) overnight at 4 °C. In order to perform western blotting analysis, the precipitated protein samples were washed with IP buffer for eight times, then resolved on SDS–PAGE for western blotting with antibodies against K48-Ub (1280 S, Cell Signaling Technology) or p53 (ab32389, Abcam).

### Immunofluorescence

Cells were grown onto glass cover slips (BD Biosciences), followed by treatment with CeO_2_ NPs for 12 h. After treatments, the coverslips were fixed with 4 % paraformaldehyde for 15 min, followed by permeabilization with 0.5 % Triton X-100 for 15 min, after which they were blocked with 5 % BSA in PBS for 1 h and then incubated with the antibody p53 (sc-126, DO-11:200 dilution) overnight at 4 °C. Goat anti-mouse IgG (Alexa Fluor 488, A-11029,1:800 dilution) was used as a fluorescent secondary antibody. The cover slips were then washed with PBS for three times and then stained with DAPI (1 µg mL^− 1^) for 10 min. Images were acquired using a confocal microscope (Nikon, Ti-E Al).

### Quantitative PCR with reverse transcription (qRT–PCR)

The cells were cultured in 6-well plate, followed by treatment with CeO_2_ NPs for 12 h. RNAiso kit (9108/9109, Takara) was used to isolate RNA from samples. Total RNA (1 µg) was reversed transcribed to cDNA according to the manufacturer’s instructions and 2 µL of the cDNA was used as a template for RT-PCR in a quantitative manner using FastStart Essential DNA Green Master (41,474,700, Roche) according to the manufacturer’s instructions at a final volume of 20 µL. Gene expression levels were normalized with GAPDH, and the average with standard deviation was presented for duplicate or triplicate experiments.

### MTT assay

The cells were plated in a 96-well plate with 10,000 cells per well followed by treatment with CeO_2_ NPs for 12 h and then cells were incubated with the MTT ( T0793-500 MG, Bio Basic) at a final concentration of 0.5 mg mL^− 1^ for 4 h at 37 °C. Finally, DMSO was added to the incubation medium to dissolve the formazan crystals, followed by shaking the plates for 15 min in the dark. An Elx800 spectrophotometer (BioTek) was used to measure the results based on the wavelengths of 490/570 nm.

### Apoptosis assay

In order to detect apoptosis in the cells, the Annexin V-FITC Apoptosis Detection Kit was used (Catalogue #C1062S, Beyo-time, China). During the experiment, the cells were cultured in 12-well plates, followed by a treatment with CeO_2_ NPs for 12 h. The cells were stained and the Annexin-positive cells were detected with flow cytometer analysis.

### Colony formation assay

The cells were cultured in 6-well plate (500 cells per well), followed by treatment with PBS or CeO_2_ NPs for 12 h. After treatments,the cells were then put into a new medium containing 10 % FBS. After being cultured for another ten days, colonies were fixed with 4 % paraformaldehyde for 20 min, stained with 0.1 % crystal violet and then washed with PBS for several times and photographed.

### Cell migration assay

The cells were cultured in 24-well plate and created a wounded by manually scraping the cell monolayer with a p10 pipet tip, and then washed twice with PBS. As a reference point for the first image acquisition, the marking on the culture dish were used as a guide, and then cells were treated either with PBS or CeO_2_ NPs for a period of 12 h. Taking the images at the time point of the migration was to quantify the migration rate of the cells to the wound.

### ROS detection

The cells were cultured in 12-well plate, followed by treatment with PBS or CeO_2_ NPs for 4 h. After treatments, cells were placed in Opti-MEM in the absence of FBS and antibiotics for 20-25 min at 37 °C staining with DCFH-DA (S0033, Beyotime, China). After being washed with sterile PBS for four times, cells were digested with trypsin, and the mean fluorescence intensity was determined using flow cytometer (BD, Bioscience). On the other hand, images were acquired using fluorescence microscopy (Nikon, DS-Fi3).

### Transmission electron microscopy (TEM)

On a copper grid coated with carbon film, 5 µL droplets of CeO_2_ NPs (200 µg mL^− 1^) were added. In order to obtain TEM images, JEOL JEM- 2100 F Transmission Electron Microscopes with a 200 kV working voltage were used.

### Zeta potential measurement

It is a simple electrochemical measurement using a disposable capillary cell (DTS1061, Malvern, UK) by vortexing 1 mg of CeO_2_ NPs in 1 mL of water,  and then measuring the zeta potential with a Malvern Zetasizer (Nano ZS90, Malvern, UK) by using a 633 nm He/Ne laser.

### DLS measurement

The size distribution of CeO_2_ NPs were measured by using a Malvern Zetasizer Nano ZS90 instrument with a He/Ne laser (633 nm) after it were resuspended in 1 mL water, placed in a square polystyrene cuvette (DTS0012, Malvern, UK), and then subjected to a size distribution analysis.

### X-ray diffraction (XRD) assay

The X-ray diffraction (XRD) patterns for CeO_2_ NPs (20 mg) powers were identified by X-ray diffractometry using Cu Kα radiation.

### Immunohistochemistry staining

Mouse tumor tissues were collected, and paraffin-embedded tissue sections were dewaxed and rehydrated in xylene and graded ethanol solution. Sections were stained with p53 antibody (sc-126,1:200 dilution). The cell nuclei were stained with hematoxylin.

### Animals

BALB/c nude mice (female, 18-20 g, 4-6 weeks) were purchased from Beijing Vital River Laboratory Animal Technology Co., Ltd. (Beijing, China). All animals were cared for in accordance with the guidelines set out in the Guidelines for the Care and Use of Laboratory Animals and are maintained at the SPF Animal Facility at South China University of Technology.

### Tumor model study

For BxPC-3 tumor model, 1 × 10^7^ BxPC-3 cells were subcutaneously injected into the right side of nude mice with 80 µL PBS mixed with 20 µL Matrigel (BD Biosciences). In order to test the effectiveness of the treatment procedure, mice were randomly assigned to two groups (5 mice in each group) and the treatment procedures were initiated when their tumor volumes reached about 100 ~ 150 mm^3^, with the indicated injections being administered twice per week into their tail veins. As part of the experiment, the following treatments were applied: PBS, CeO_2_ NPs (30 mg kg^− 1^). The body weight and tumor size of mice were measured every 3 days. The mice were sacrificed on the 15th day of treatment, and the excised tumors were weighed and photographed.

### Pharmacokinetic study

Female BALB/c nude mice were randomly divided into two groups and were given intravenously CeO_2_ NPs at a dose of 30 mg kg^− 1^. The method of this test was carried out according to the previous report. In simple terms, a blood sample was taken from the posterior orbital mass of the eye, then placed in the heparin tube, and plasma was collected by centrifugation at predetermined time points (0, 1, 3, 7, 11, 24, and 36 h). Take the same amount of plasma and nitrate the plasma samples. The contents of Ce in plasma were determined by ICP-MS, and the time values at each time point were normalized to 0.

### Statistical analysis

A *student’s t-test* was performed to analyze the data, with all results expressed as Mean Standard Error of Mean. *p < 0.05, **p < 0.01 and ***p < 0.001 were considered statistically significant.

## Electronic supplementary material

Below is the link to the electronic supplementary material.

Supplementary Material 1

## Data Availability

All data generated or analyzed during this study are included in this article.
