# Supplementary material for: Precise pancreatic cancer therapy through targeted degradation of mutant p53 protein by cerium oxide nanoparticles
Source: J Nanobiotechnology. 2023 Apr 1;21:117. doi: 10.1186/s12951-023-01867-6 (PMC10067194; doi:10.1186/s12951-023-01867-6)
Supplement: Supplementary file 1 — Supplementary Material 1 [file 12951_2023_1867_MOESM1_ESM.docx]

**Supporting Information**

**Precise pancreatic cancer therapy through targeted degradation of mutant p53 protein by cerium oxide nanoparticles**

Hao Zhang^1^, Wang Zhang^1^, Bochuan Hu^2^, Xiaohua Qin^2^, Tianxiang Yi^1^, Yayi Ye^2^, Xiaowan Huang^2^, Yang Song^1^, Zhenyu Yang^1^, Jieying Qian^2,3*(🖂)^, Yunjiao Zhang^1,2,3*(🖂)^

^1^ School of Medicine, South China University of Technology, Guangzhou, 510006, P. R. China.

^2^ School of Biomedical Sciences and Engineering, South China University of Technology, Guangzhou International Campus, Guangzhou, 511442, P. R. China.

^3^ National Engineering Research Center for Tissue Restoration and Reconstruction and Key Laboratory of Biomedical Engineering of Guangdong Province, South China University of Technology, Guangzhou, 510006, P. R. China;

^*^ Correspondence and requests for materials should be addressed to YJ Zhang (email: [zhangyunjiao@scut.edu.cn](mailto:zhangyunjiao@scut.edu.cn)) or JY Qian (email: qianjieying1314@scut.edu.cn)


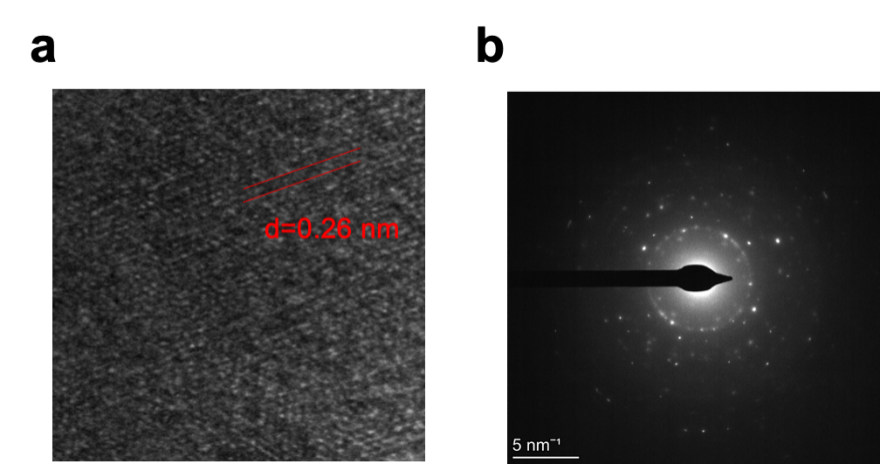


**Figure S1.** **(a)** HRTEM image of CeO_2_ NPs. **(b)** Selected area electron diffraction (SAED) pattern of CeO_2_ NPs.


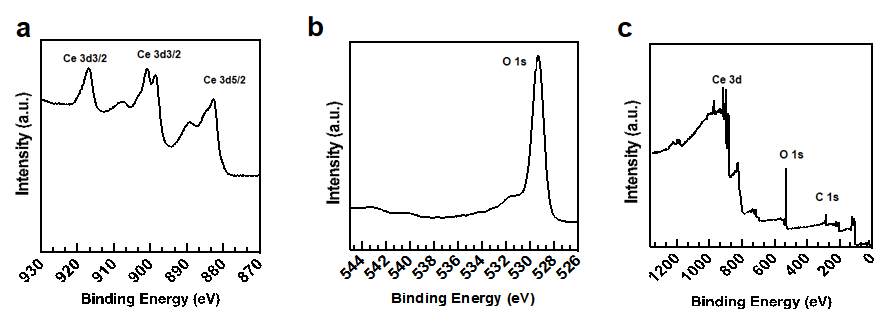


**Figure S2.** XPS spectra of CeO_2_ NPs: **(a)** Ce 3d. **(b)** O 1s. **(c)** XPS survey spectra.


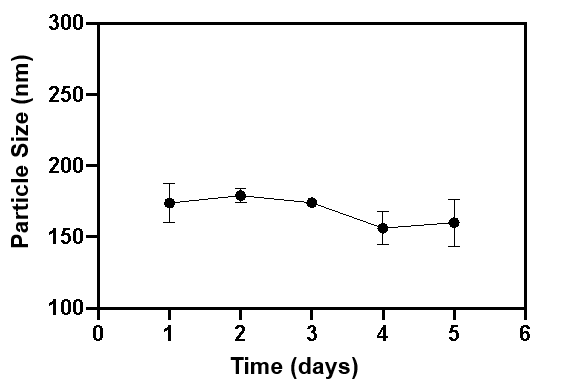


**Figure S3.** CeO_2_ NPs were evaluated for their stability in the water by measuring their particle size for indicated time.


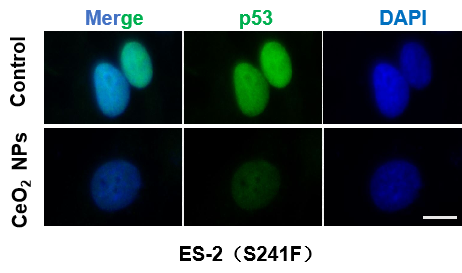


**Figure S4.** Confocal microscopic images of ES-2 cells treated with PBS (Control) or CeO2 NPs (6 μg mL^−^1) for 12 h, followed by immunostaining with anti-p53 antibody and nucleus staining with DAPI. Scale bar, 10 μm.


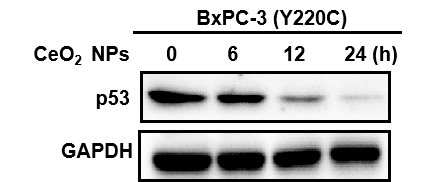


**Figure S5.** A western blotting was performed to determine the level of mutp53 in BxPC-3 cells treated with CeO_2_ NPs (6 μg mL^−1^ )  for the indicated time .


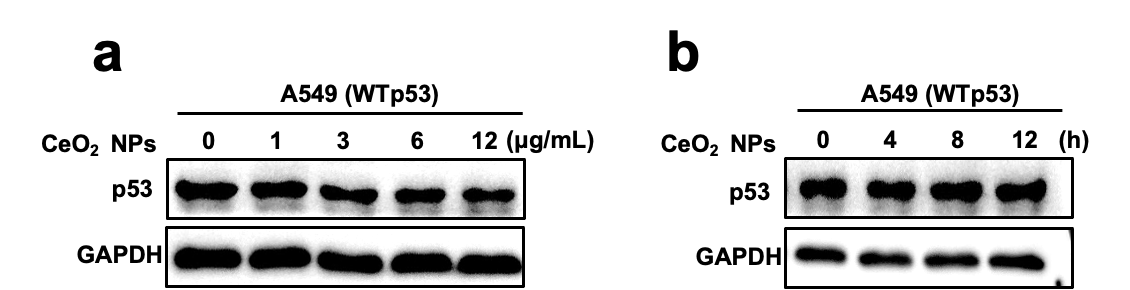


**Figure S6.** Western blotting of p53 levels in A549 cells after treatment with different concentrations of CeO_2_ NPs **(a)** for a period of 12 hours or **(b)** treatment with CeO_2_ NPs (6 μg mL^−1^) for the indicated time.


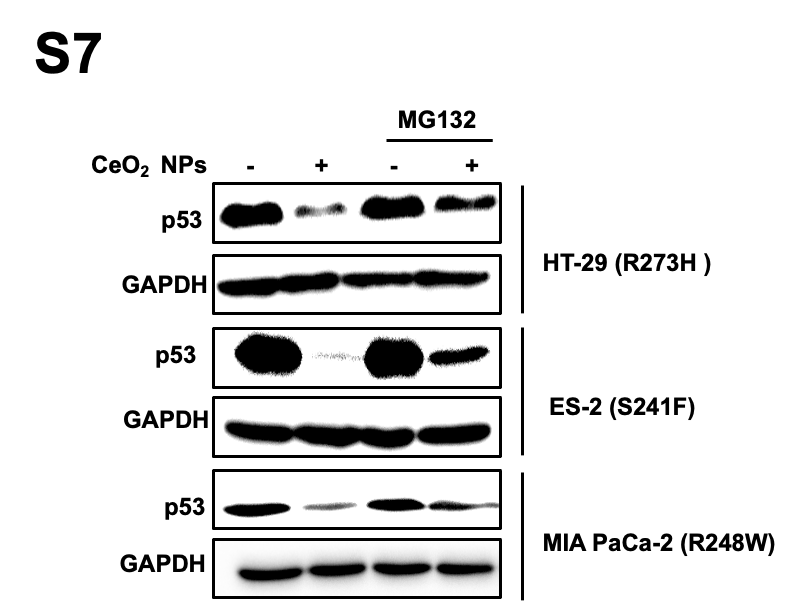


**Figure S7.** Western blotting analysis of the level of mutp53 in cells ( HT-29, MIA PaCa-2 and ES-2) after indicated treatment for 12 h. Dosing: CeO_2_ NPs (6 μg mL^−1^); MG132, 10 μM.


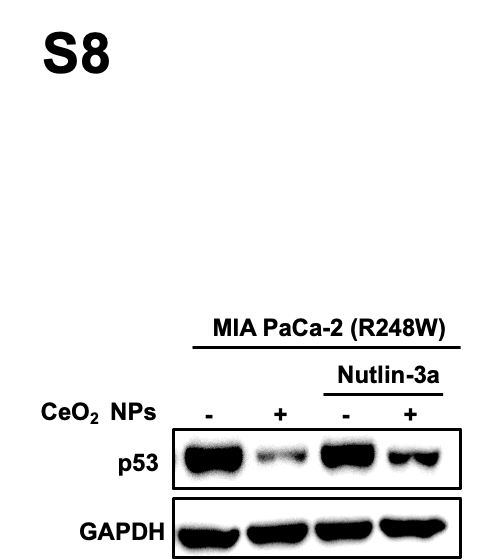


**Figure S8.** Western blotting analysis of the level of mutp53 in MIA PaCa-2 cells after indicated treatment for 12 h. Dosing: CeO_2_ NPs, 6 μg mL^−1^; Nutlin-3a, 10 μM.


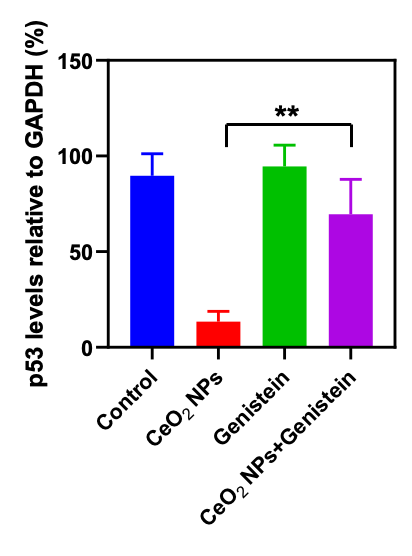


**Figure. S9.** Relative p53 levels in BxPC-3 cells treated with PBS, CeO_2_ (6 μg mL^-1^) Genistein (50 μM) and Genistein+CeO_2_ for 12 h. Mean ± s.e.m. n=3. *Student’s t-test*.


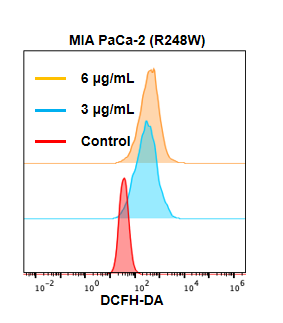


**Figure S10.** The levels of intracellular ROS were detected with DCFH-DA and analyzed by flow cytometer after treatment with PBS (Control) or CeO_2_ NPs (3 or 6 μg mL^-1^) for 4 h.


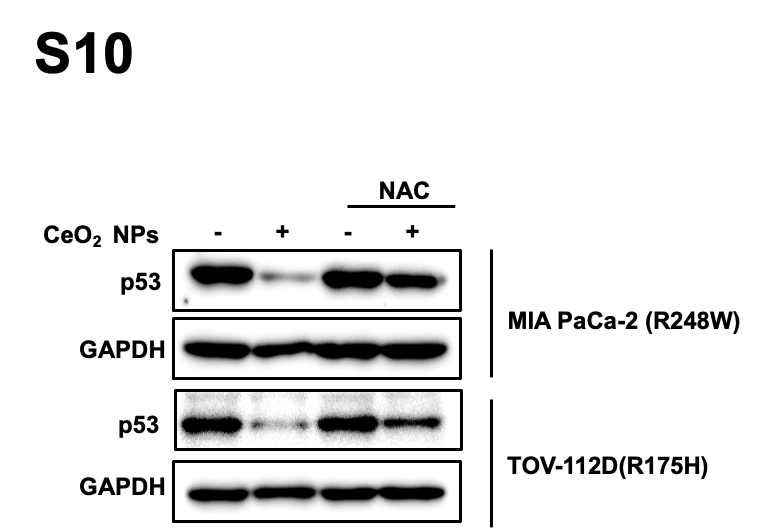


**Figure S11.** Western blotting analysis of mutp53 levels in cells (MIA PaCa-2 and TOV-112D) after indicated treatment for 12 h. Dosing: NAC, 5 mM; CeO_2_ NPs,6 μg mL^-1^.


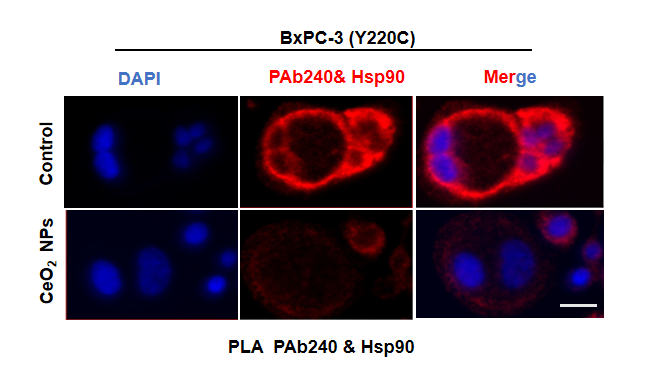


**Figure S12.** BxPC-3 cells were treated with CeO_2_ NPs (6 μg mL^-1^) for 12 h before processed for PLA assay between Hsp90 and mutp53. The cell nucleus were stained with DAPI. The images were obtained by confocal microscope. Scale bar, 10 μm.


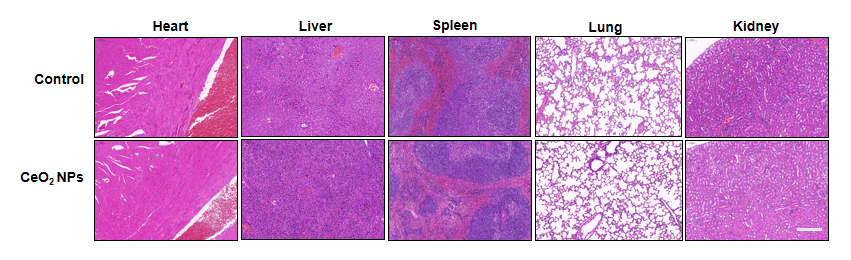


**Figure S13.**The staining with hematoxylin and eosin (H&E) of the major organs of mice were performed after treatment with PBS (Control) or 30 mg kg^-1^ CeO_2_ NPs for 24 h. Scale bar, 100 μm.


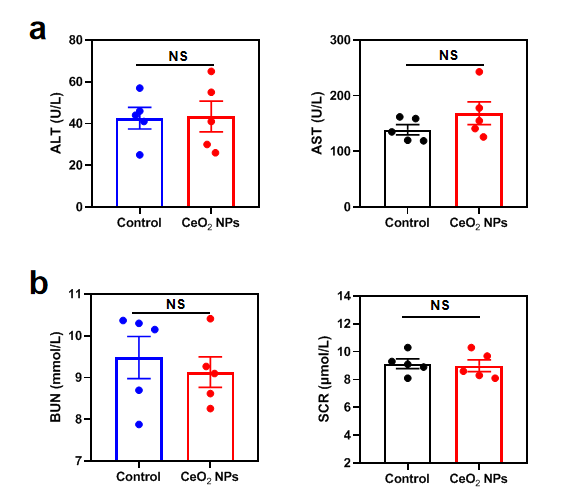


**Figure S14.** Serum levels of ALT (Alanine aminotransferase), AST (Aspartate aminotransferase), BUN (Blood urea nitrogen) and SCR (Serum creatinine) in mice after treatment with PBS (Control) or 30 mg kg^-1^ CeO_2_ NPs for 24 h. Mean ± s.e.m. n=5. *Student’s t-test*. NS: not significant.


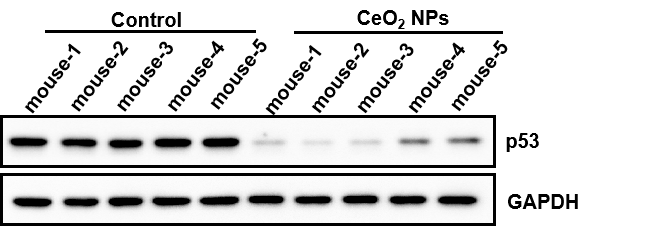


**Figure S15.** Western blotting of p53 and GAPDH for each of the excised tumor tissues in the various treatment groups of the BxPC-3 mouse model.
